# Diverse human dimensions affect the management of public and animal health impacts of free-roaming dogs in Australia: a One Health solution

**DOI:** 10.3389/fvets.2025.1666111

**Published:** 2025-10-23

**Authors:** Peter J. S. Fleming, Benjamin L. Allen, Guy Ballard, Linda Behrendorff, Andrew W. Claridge, Matthew N. Gentle, Lana Harriott, Donald W. Hine, David J. Jenkins, Brooke P. A. Kennedy, Lynette J. McLeod, Paul D. Meek, Grace Proudfoot, Nicole Schembri, Deane Smith, Jessica Sparkes

**Affiliations:** ^1^Ecosystem Management, School of Environmental and Rural Science, University of New England, Armidale, NSW, Australia; ^2^Vertebrate Pest Research Unit, New South Wales Department of Primary Industries and Regional Development, Orange, NSW, Australia; ^3^Centre for African Conservation Ecology, Nelson Mandela University, Port Elizabeth, South Africa; ^4^Vertebrate Pest Research Unit, New South Wales Department of Primary Industries and Regional Development, Armidale, NSW, Australia; ^5^Queensland Government Department of Environment and Science, Queensland Parks and Wildlife Service, K’gari, QLD, Australia; ^6^Vertebrate Pest Research Unit, New South Wales Department of Primary Industries and Regional Development, Queanbeyan, NSW, Australia; ^7^School of Science, University of New South Wales, Canberra, ACT, Australia; ^8^Pest Animal Research Centre, Biosecurity Queensland, Department of Primary Industries, Toowoomba, QLD, Australia; ^9^School of Psychology, Speech and Hearing, Faculty of Science, University of Canterbury, Christchurch, New Zealand; ^10^School of Animal, Environmental and Veterinary Sciences, Charles Sturt University, Wagga Wagga, NSW, Australia; ^11^Vertebrate Pest Research Unit, New South Wales Department of Primary Industries and Regional Development, Coffs Harbour, NSW, Australia; ^12^One Health Unit, interim Centre for Disease Control, Department of Health, Disability and Ageing, Canberra, ACT, Australia; ^13^Vertebrate Pest Research Unit, New South Wales Department of Primary Industries and Regional Development, Yanco, NSW, Australia

**Keywords:** commensal, dingo, environmental psychology, native fauna, peri-urban, remote, typology, wild-living dog

## Abstract

The socio-ecological roles and status of free-roaming dogs (*Canis familiaris*) in Australian urban, peri-urban and other environments are complex. We review and synthesise those complexities and identify knowledge deficits and impediments to adoption of best-practice management of free-roaming dogs. Briefly, perceptions of the roles and impacts of free-roaming dogs in Australia are affected by their status as native, introduced and culturally significant animals, the situations in which they occur and the other species, including humans, with which they interact. Their negative, neutral and positive impacts often occur contemporaneously making free-roaming dogs a ‘wicked’ problem. We propose and evaluate a One Health-based solution using an environmental psychology perspective in a strategic adaptive management framework. This includes: a typology of free-roaming dogs that assists in the situational definition of animal and public health and welfare issues; identification of some human dimensions affecting management of free-roaming dogs; identification of discipline specialities that require inclusion in an effective One Health approach; audience segmentation, and; priorities for research and policy development to encourage adoption of best-practice management for each occurrence of free-roaming dog impacts.

## 1 Introduction

Dogs (*Canis familiaris*) have important roles in historical and modern societies and ecosystems across the world. They were likely the first animal species to be domesticated (1), being derived from the Grey Wolf (*Canis lupus*) (2) or a common ancestor (3) by accidental/self-domestication [*sensu* (4, 5)] and purposeful domestication and breeding processes10–33 thousand years ago (1, 6, 7), and some have feralised (8). They have a global distribution corresponding with that of humans and have an estimated population size of ~986,900,000–1,086,900,000 (9). The spatio-temporal overlap of free-roaming dogs and humans leads to ongoing conflict for resources and contact points for pathogen transmission and aggressive encounters (10–12).

**Figure 1:**
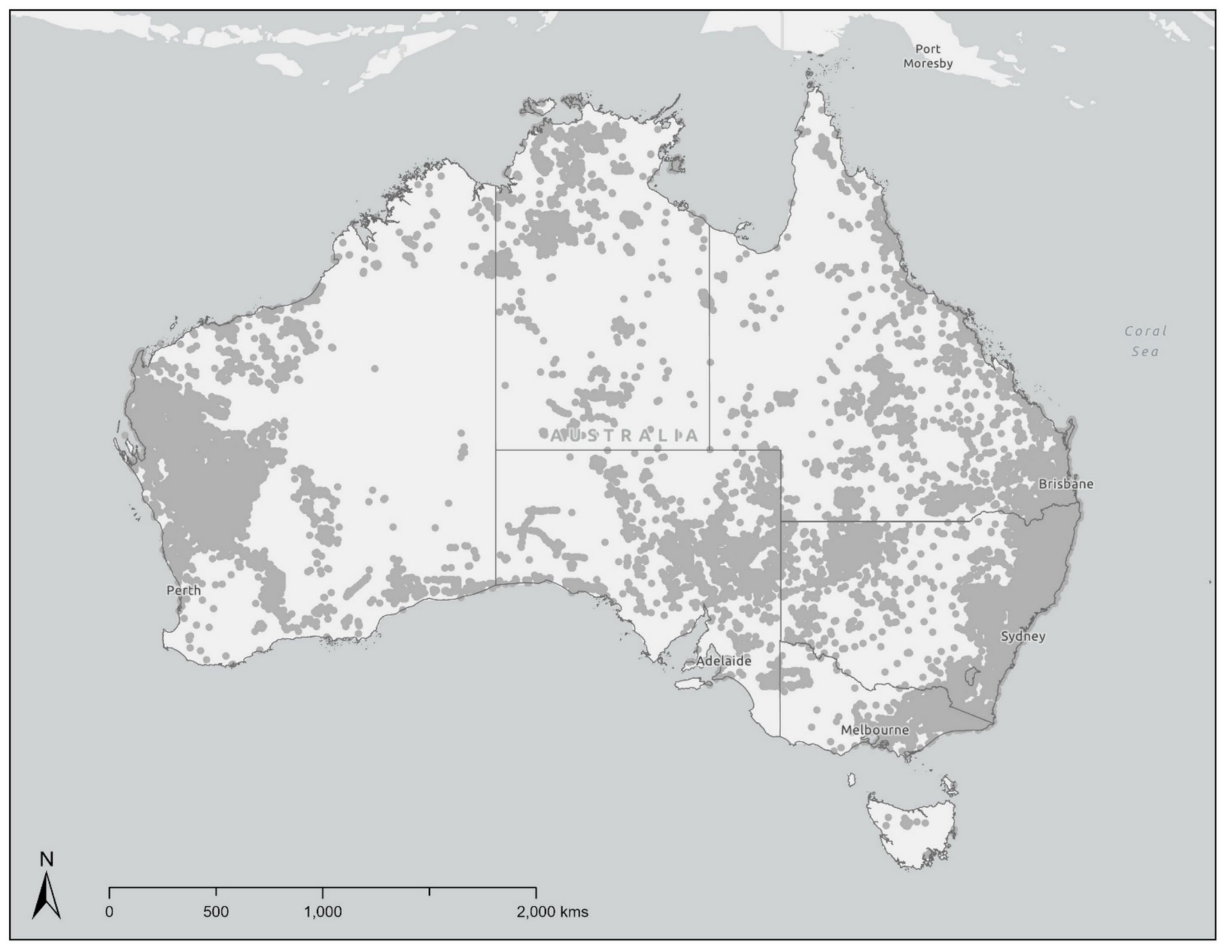
Point data indicating the distribution of free-roaming dogs in Australia (*n* = 75,580). Data sourced from FeralScan: WildDogScan (*n* = 56,876); Atlas of Living Australia (*n* = 14,337); and Stephens, D. and Fleming, P.J.S. unpublished wild dog genetics study data (*n* = 4,367), in the period 01/01/2010–30/04/2025. Data are displayed is limited to presence-only data, and absence of data does not indicate absence of free-roaming dogs. Urban and restrained dogs have been omitted from the combined dataset.

Free-roaming dogs in most countries are usually feral modern breeds and mongrels that are commensal (9), but those in Australia, New Guinea and eastern Indonesia comprise: naturalised ancient breeds that have been feral^1^ for several thousand years and are sometimes considered native [i.e., singing dogs and dingoes (13, 14)]; modern dog breeds that have been more recently introduced; and cross-breeds of ancient and modern breeds that have been purposely or accidentally incorporated into the free-roaming dog gene pool (13, 15). There are between 3.1 and ~6.3 million owned dogs in Australia (9, 16), some of which roam freely (17, 18). Additionally, there are likely >50,000 dingoes and other wild-living dogs extant across most ecosystems on the mainland (19) and recorded densities range from 0.01 dingoes km^−2^ to 8.0 dingoes km^−2^ (20–22): dingoes never reached Tasmania or Kangaroo Island, but feral modern dog breeds have been recorded in Tasmania (Figure 1). The genetic diversity and nomenclature of wild-living dogs is contentious and currently strongly debated (23–28), as are the ecological roles they fulfil (29–31), but we will not focus on those conflicts here. Nevertheless, because of their multifarious roles and occurrences, free-roaming dogs in Australia are an extreme example of human-wildlife conflict and are a wicked issue (32, 33). We will instead concentrate on Australia and the issues of free-roaming dogs in different contexts, regardless of their genetics or phenotype.

Australia is a federation of six states and two territories, all of which have different and often conflicting legislation and policy relating to the management of free-roaming dogs. This adds to the complexity of status and management options and can compound policies and actions relating to public and animal health and welfare. Most states and territories have legislations pertaining to the keeping of dogs (e.g., New South Wales *Companion Animals Act 1998* No 87 and Australian Capital Territory *Domestic Animals Act 2000*) and Acts to inhibit cruelty to animals (e.g., various *Prevention of Cruelty to Animals* Acts and Northern Territory *Animal Protection Act 2018*), which often incorporate regulations about the responsibilities of owners towards their dogs and the community. While there is often continuity among those state and territory regulations and objectives for managing dogs for the benefit of the animals and their humans, legislation on wild-living dogs is much more diverse and management capacity varies accordingly across jurisdictions. The status of the dingo component of wild-living dog populations also varies between the Australian Government and state and territory legislatures: for example, under the Australian Government *Environmental Protection and Biodiversity Conservation Act 1999*, Section 528, dingoes are classed as a “native species” deserving conservation because they were extant prior to 1,400 CE, but the dingo is explicitly excluded from the New South Wales *Biodiversity Conservation Act 2016*, which uses 1788 CE as the cut-off date for native animals. The duality of status and controversy about roles of wild-living dogs in Australian ecosystems also causes conflict that impedes concentration on their animal and public health impacts. Because free-roaming dogs can cause agricultural damage by variously impacting upon livestock and can have adverse effects of wildlife, biosecurity acts prevail across Australia (e.g., South Australian *Dog Fence Act 1947*, Queensland *Biosecurity Act 2014*, and Western Australia *Biosecurity and Agriculture Management Act 2007*), that outline land owners’ and occupiers’ responsibilities to restrict adverse impacts of free roaming dogs.

While free-roaming dogs in Australia pose significant challenges to wildlife conservation, agricultural interests, and public health, they fulfil important social and cultural roles. Addressing this multifaceted issue requires a One Health or One Welfare approach. Globally, One Health is an initiative led by the Quadripartite that includes the World Health Organisation (WHO), Food and Agriculture Organisation of the United Nations (FAO), the United Nations Environment Programme (UNEP) and the World Organisation for Animal Health (WOAH), that recognizes the interconnectedness of people, animals and environment on the health of all three. The One Health High-Level Expert Panel (OHHLEP) defined One Health as “an integrated, unifying approach that aims to sustainably balance and optimize the health of people, animals and ecosystems” (34). This statement is all-encompassing, seeking to mobilise numerous cross-disciplinary sectors and community at all levels, to come together to advance wellbeing and tackle threats to health and ecosystems. Although there are tools to enable collaboration and multilingual eLearning guides, and provide software connections for health threat reporting, the mechanisms to enable integration and bottom-up acceptance and One Health action (e.g., the Toolkit: One Health Surveillance Toolkit) are purposefully vague or high level. Both the definition and current tools seek to provide a framework to be considered as an overarching set of guiding principles that can be tailored to specific stakeholders and opportunities or circumstances (34). One Welfare (35, 36) is a similar concept and an extension of One Health. It is more specific about wellbeing of animals, humans and their environments, encapsulates mental and physical wellbeing and acknowledges that animal welfare depends on and influences human welfare and environmental sustainability (36). However, both initiatives are unspecific about the linkage between the conceptual and the practical on-ground application.

The fundamental requirement for adoption of One Health or One Welfare approaches to free-roaming dog impacts on human and other animal health and wellbeing in Australia is human behaviour change. Raising awareness through education and extension can be a useful, often essential, step towards behaviour change but these are insufficient in themselves (37). Taking an environmental psychology design to an issue helps to change peoples’ behaviour by targeting messages to audiences in an experimental framework that enables measurement of adoption and iterative improvement (38). There are four guiding principles to such an approach: a focus on human behaviour and measuring current behaviours in relation to an issue; understanding factors that drive or impede the adoption of desired behaviours; aligning the interventions to these factors and determining the messages that will influence different segments of the audience to change their behaviour; and measuring change in response to the interventions (37, 39). Applying adaptive management to that framework enables iterative changes to interventions to match the changing audience; this is the strategic approach in invasive species management [e.g., (40, 41)].

Our objectives here are to review the issues relating to the conflict about managing free-roaming dogs in Australia, and evaluate them in a One Health context. Specifically, we:

- identify naming and taxonomy issues and propose a typology of Australian free-roaming dogs that emphasises the situations in which they occur;
- identify the human dimensions affecting management of the different types and situations
- identify what the public and animal health and welfare impacts are and the different situations where those impacts occur;
- propose and appraise best practices in relation to implementation of a One Health approach;
- discuss the applicability of an integrated One Health approach to managing public and animal health impacts of free-roaming dogs;
- propose the use of environmental psychology and a strategic adaptive management approach to achieve iterative behaviour change necessary for adoption of the holistic concepts, and;
- identify the knowledge and practice deficits that impede progress towards such integrated approaches.

## 2 Typology of Australian free-roaming dogs

Typologies of free-roaming dogs have been provided for different purposes. In Australia, we have dingoes and other wild-living, unrestrained dogs, but their status and responses to them are varied (42–44). Part of the wicked problem of managing wild-living and other free-roaming dogs in Australia resides in the definitions we use for them. Language is important and different names elicit different responses from people, depending on their understanding, world views, attitudes and feelings towards different types of dog, and the different situations in which they occur. Here, we provide a typology for wild-living dogs for Australian contexts to remove some of the ambiguity surrounding their names and consequent status.

Most people are familiar with owned, modern domestic dogs, which have many values, and have attitudes towards and responses to them when they are constrained or managed by owners (45–47). The contexts in which dogs occur are “contained” (restrained or owned), and a continuum of “unrestrained” or free-roaming dogs, some of which are also owned either by individuals or communally. The former is self-explanatory (see type 1 below), but in practice restrained dogs are either always in the company of humans or domiciled with people such that their movements are restricted, i.e., housed, chained or confined to a run or yard. Contained dogs (primarily pets, but also zoo and research animals, Type 1 below) stand alone—they are usually not free to roam and subject to veterinary supervision and so make relatively limited contributions to the health of other dogs, humans, wildlife, livestock and domestic animals compared to free-roaming animals. However, one-way interactions can occur where free-roaming dogs contact contained animals, either directly (e.g., copulating with chained animals) or indirectly, e.g., visitation of restrained dogs on leads to common-use faecal or urine deposit sites.

Any dog that is free-to-roam (i.e., “free-roaming”), albeit temporarily (i.e., short-term strays, Type 2a below), is functionally different to contained dogs; they have much higher chances of affecting, or being affected by, other organisms. The extent to which they move (spatial scale) and the frequency with which they interact with other dogs (linked to, but not solely reliant upon density) will necessarily also affect their probability of involvement in health and welfare scenarios.

**Figure 2:**
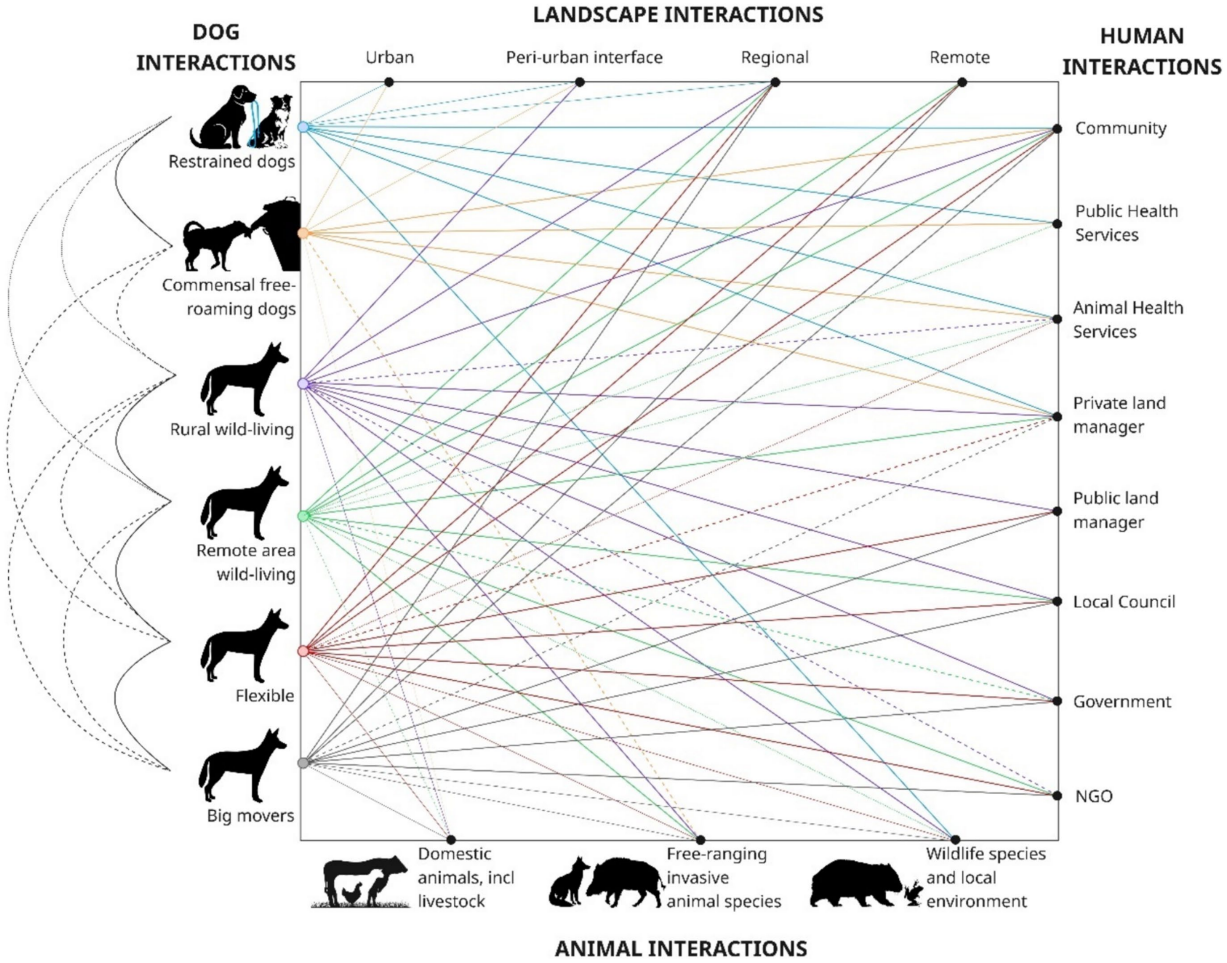
The interactions of different types of dog across landscapes, and with people and other animals, showing relative dependence on anthropogenic resources (line density indicates relative strength of interactions). Community includes indigenous and non-indigenous communities and community services; Public health services includes general practitioners and allied health workers; Animal health services includes veterinarians; private land manager includes landowners and leasers, including livestock producers and indigenous land manager; Public land manager includes national park estate, forestry estate and crown lands; and NGO includes non-government organisations such as RSPCA, Animal Management in Rural and Remote Indigenous Communities, Wildlife Health Australia and Wildlife Information, Rescue and Education Service.

Uncontained dogs fit into five free-roaming types over a continuum from partly-restrained to completely wild-living (see categories 2–6 below and Figure 2). These delineations are based on observations and movement data from studies undertaken across Australia by the authors and others [e.g., (18, 48–53)]. The types of dog are:

1 Contained modern dogs (synanthropic, hyper-abundant, focal resources);
2 Commensal free-roaming dogs (synanthropic, variable abundance, using focal resources, refuse tips, town fringes and parks and green spaces) including:
 a Urban wanderers (owned, hyper-abundant, making occasional forays away from their residence, exploiting diffuse resources);
 b Short-term strays (owned, hyper-abundant, making frequent forays, e.g., overnight, away from their residence, diffuse resources);
 c Long-term strays (unowned or community-owned modern dogs with no residence, less abundant, using diffuse or focal resources);
 d Urban wild-living dogs (unowned, less abundant, using diffuse or focal resources);
 e Peri-urban wild-living dogs (unowned, variable abundance, using diffuse or focal resources);
 f Remote wild-living dogs (unowned, variable abundance but locally hyper-abundant, using diffuse and focal anthropogenic food and water resources, e.g., mining camps, bores), and;
 g Urban/peri-urban visitors (foray takers into urban and peri-urban environments from rural and remote areas)
3 Rural/regional wild-living dogs—often living across tenures (unowned, variable abundance, using diffuse agricultural and environmental resources);
4 Remote wild-living dogs—often living on extensive single tenure in extensive agriculture, conservation and indigenous-managed landscapes (generally at low density but occasionally locally hyper-abundant, e.g., in response to rabbit or rodent plagues or macropod hyperabundance, mostly using diffuse resources);
5 Flexible—wild-living dogs that either change their situation during their lifetime (e.g., changing from peri-urban to rural/ regional) or move frequently between rural or remote area and commensal categories [e.g., peripatetic dingoes (48)], and;
6 Big movers—rural/ regional or remote wild-living dogs that move extensive distances across tenures [e.g., (54)]

As an individual dog’s reliance on human resources and their scale of movement changes, they can conceivably move between ‘types’ of dog (e.g., between commensal Types 2 and flexible Type 5). However, in the absence of empirical evidence, the specific thresholds between ‘types’ are necessarily uncertain.

Our typology attempts to categorise free-roaming dogs based on their situation without hindering interpretation of legislation and policy instruments, while encouraging One Health and One Welfare approaches. For example, the New South Wales *Companion Animals Act 1998 No. 87* has the objectives of promoting responsible ownership of cats, dogs and other companion animals, and preventing harm to companion animals, other animals and people, but does not include harm to wildlife, ignores free-roaming wild dogs defined under the *New South Wales Biosecurity Act 2015* and takes little account of First Nations cultural obligations and customs. The *Act* requires that companion dogs be identified by 12 weeks of age, registered with a local authority by 6 months of age and be kept from defecating in a public place and escaping from the premises where it is domiciled. Owned dogs that habitually wander or escape or repeatedly defecate on property other than its normal residence are considered “nuisance dogs” under the *Act*. Similar requirements apply to owners of dogs in Queensland under the Queensland *Animal Management (Cats and Dogs) Act 2008*. Working dogs and some other dogs are exempt from registration and identification under both Acts.

We highlight the issues of free-roaming dogs at remote mining camps because they are a special case. Type 2f, 4 and 5 dogs predominate, co-existing with fly-in-fly-out itinerant residents living in rotation in infrastructure that resembles a town, but which lacks all the usual municipal facilities and permanent residents. Co-existence of workers and dogs in camps and associated facilities is often addressed through management plans, and include prohibition of workers’ dogs on site, the discouragement of dog feeding and interactions that create non-wild behaviours in dogs, and containment of refuse to exclude dogs. These free-roaming dogs can be partitioned into three main categories (48, 50); those that maintain a wild existence (Type 4), those that fluctuate between human resource use and wild existence (Type 5), and those that reside in camps (i.e., Type 2f) and have returned to their domestic origin behaviours (48). The latter group present challenges for mining companies because the dogs become familiarised with humans, human-dependent, brazen and pests that have no fear of humans and have attacked workers.

### 2.1 Movement behaviour of free-roaming dogs

**Figure 3:**
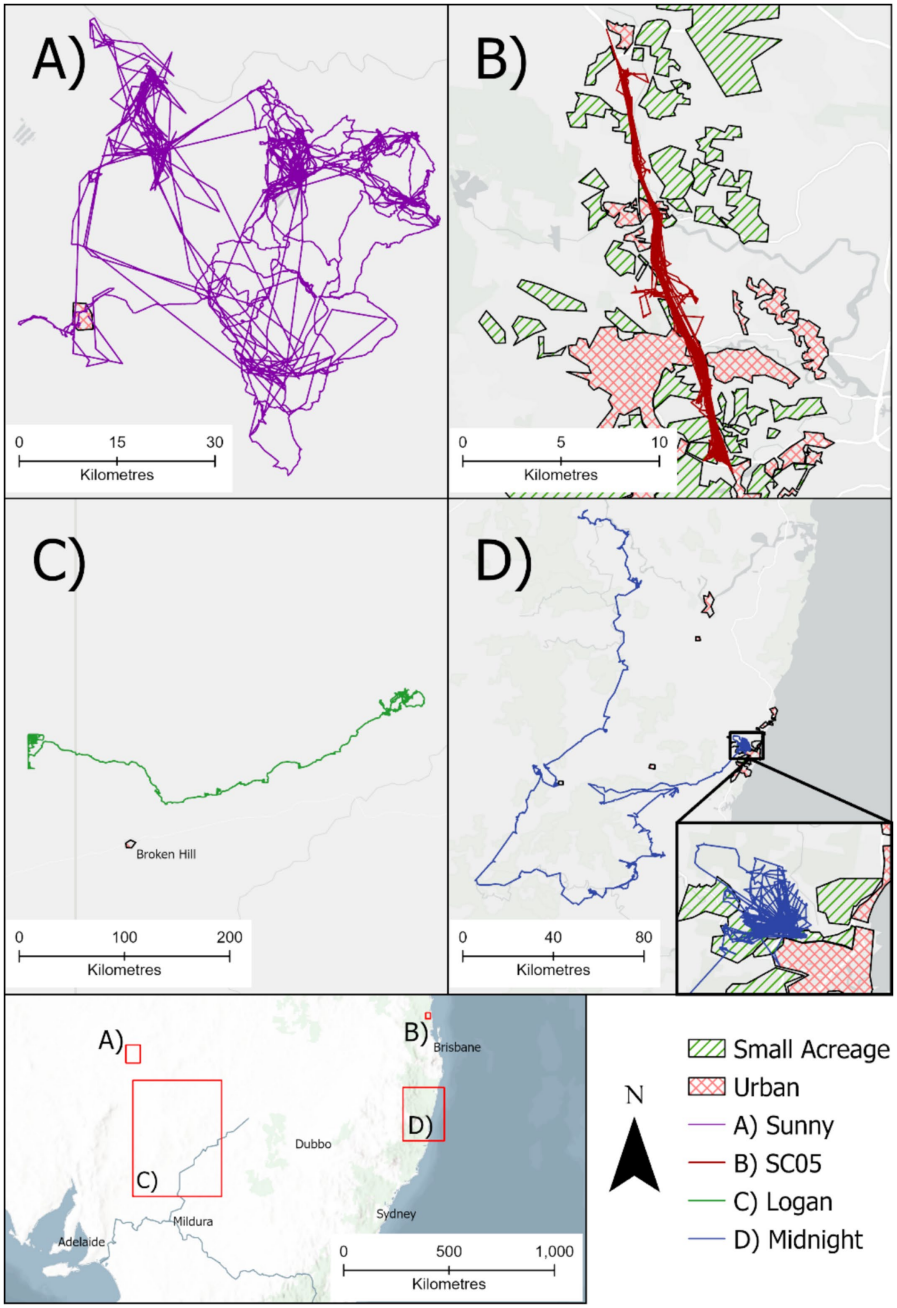
Examples of the forays of wild dogs of different typologies tracked with GPS collars. See text for explanations. Red hashed polygons show heavily urbanised areas, green diagonal polygons show areas with small acreage habitations, all else is larger rural properties, bush land, remote or unknown land uses. Scales vary between panels.

Key to identifying hazards of free-roaming dogs to other animals and people is their movement behaviour, which we have attempted to encapsulate in our typology. In Figure 3, four different types of dog are presented to demonstrate the complexity of situations and likelihoods of interaction and hence different disease and attack risks for people, wildlife, livestock and domestic animals.

A male Type 5 free-roaming dog (Sunny, Figure 3A) was trapped and released at a waste facility offering constant food and water near a remote mine site in South Australia, but mostly utilised a large area of the surrounding arid landscape (48). Free-roaming dogs of dingo parentage also use areas with dense human populations. For example, a female Type 2f free-roaming dog trapped and released on the Sunshine Coast in Queensland (SC05, Figure 3B) spent the tracking period travelling along a ~24 km stretch of the Bruce Highway between heavily urbanised areas (52). Interstate movements occur, such as the male Type 6 free-roaming dog (Logan, Figure 3C) who was initially trapped site in a semi-arid rural rangeland environment in north-western New South Wales and travelled ~500 km during a 2 weeks period to a remote arid environment in South Australia (Smith et al. in review). An example of a Type 5 free-roaming dog is provided by a male wild dog (Midnight, Figure 3D) that spent ~3 months post-release in a peri-urban environment outside of the coastal resort town of Coffs Harbour, New South Wales. It then undertook a 3-month foray into rural areas, first in coastal hinterland to the south of Coffs Harbour, then up onto the Great Dividing Range in the Northern Tablelands before returning to North Coast hinterland north-west of Grafton, New South Wales, where the GPS collar dropped off ~120 km from its point of capture (Meek et al. unpublished data).

## 3 Australian human dimensions of free-roaming dogs

The close relationship dogs have had with humans over millennia (55–57) has resulted in diverse cultural perspectives and attitudes towards dogs. Different people and cultures treat and value dogs and different types of dogs differently. These perspectives, attitudes and values can also change over time. For example, some Islamic communities view dogs as unclean animals and discourage their keeping. Some Buddhist communities value dogs highly and regularly provide supplementary food to stray dog populations, facilitating uncontrolled dog population growth. Many Judeo-Christian communities have a ‘love-hate relationship’ with dogs, where modern owned dogs are valued highly and treated compassionately (58) whereas stray or unowned dogs less so. Polytheistic, indigenous societies and cultures around the world similarly exhibit varied relationships with dogs (46). This diversity of perspectives, beliefs and attitudes towards dogs means that it is impossible to be certain that a given society, culture, religion or group has a consistent attitude towards dogs or dog management. Australia, as a multicultural society, reflects the multitude of perspectives towards free-roaming dogs.

### 3.1 Humans love dogs

Many humans have a deep-seated affection for dogs, a bond that has evolved over thousands of years (59). This relationship is usually mutually beneficial, significantly enhancing the quality of life for both humans and dogs. Historically, dogs have played a variety of roles in human societies. They have been loyal companions, providing emotional support and companionship. They hold cultural significant symbolism and kinships with indigenous communities. As protectors, dogs have guarded homes and livestock, ensuring safety and security. Additionally, dogs have served as working animals, assisting in hunting, herding, detection, therapeutic support and search-and-rescue operations. This multifaceted relationship highlights the deep connection and interdependence between humans and dogs, showcasing how integral they have become to human lives (60, 61).

Humans have an innate tendency to seek connections with nature and other forms of life, including dogs, and this relationship is evident in the way dogs are often considered part of the family, providing emotional support and companionship (62). Dogs can serve as a source of social support, helping to mitigate feelings of loneliness and isolation, especially in urban environments where human interactions may be limited (63). Dogs can act as social catalysts, encouraging interactions between people in public spaces such as parks, fostering a sense of community, stronger social networks and improved mental health (64). Beyond that, dogs can have true working relationships with humans. For example, detector dogs are used to assist in pinpointing human diseases, weapons and chemicals in the environment (65), and locating gourmet truffles in truffieres and natural forests (66).

The presence of a dog can create a calming effect, reducing anxiety and promoting a sense of security. Many people report feeling happier and less stressed in the presence of their dogs, which can be attributed to the release of oxytocin, a hormone associated with bonding and affection (67). The simple act of petting a dog has been shown to lower blood pressure and heart rate, contributing to overall wellbeing (68, 69). Dogs have also been used in therapeutic settings, such as in animal-assisted therapy, where they help individuals with mental health issues, physical disabilities, or chronic illnesses (70).

Dogs also gain numerous benefits from their relationship with humans, which contribute to their overall improved welfare, wellbeing and reproductive success (63, 71, 72). Dogs, like humans, are social animals and thrive on interaction and the affection they receive from their human companions. Domesticated dogs (Types 1, 2a, 2b and 2c) receive protection from predators and harsh environmental conditions. When properly cared for, they have a safe place to live, a consistent food supply, and receive regular physical and mental activities and veterinary care, which can significantly extend their lifespan. Positive interactions with humans can also reduce stress and anxiety in dogs (67, 73).

A likely driver for contemporary attitudes towards free-living dogs is the increasing embrace of dogs into non-indigenous family kin relationships. Social media and a solid campaign by veterinarians and veterinarian suppliers in Australia to encourage pet ownership akin to long standing behaviours by Europeans has impacts on dog management. Only 50 years ago a pet dog was just that, a pet dog. Rarely were they an inside animal, most were a friend who lived a dog’s life outside, still loved by their owners but the relationship was owner and pet. Possibly because of increased human population growth and density and the increase in single person households, a change in behaviour in Australian society has seen dogs becoming more a part of the human family, often no longer dogs but children or “fur babies.” Relationships with dogs are replacing human relationships (45), and dogs are permitted in places previously prohibited like shopping centres, cafes, breweries and hardware stores. They can also dominate the flavour of social media (74) and drive behavioural acceptance and change in society. The consequences of this shift from dogs as pets towards dogs as humans is influencing social license where dog management is required, e.g., after attacks on humans. The lines have become blurred between wild-living dogs and modern domestic dogs, some have described it as a social construct (75) that is causing conflict between the general public and land managers who are required to manage attacks on livestock and wildlife, as well as disease spread.

The strong relationship between humans and dogs creates significant challenges in managing free-roaming dogs. One major issue is the difficulty in distinguishing between different types of dog in the field, making it hard for even well-intentioned individuals to report dog-related damage accurately (76). This poor discrimination can lead to dangerous interactions, as people may mistakenly approach wild dogs thinking they are pets. Additionally, the deep affection many people have for dogs makes them hesitant to report free-roaming dogs, knowing that captured animals might be euthanised (76, 77). Furthermore, there can be a reluctance to use traps or poison against type 4 and 5 dogs because of the hazards they might pose to contained and commensal dogs and other pets, complicating efforts to manage the abundance of free-roaming dog populations effectively (76–79).

### 3.2 Dingoes and aboriginal communities—kinship and totems

Dingoes and dogs are important to peoples of all Australia’s First Nations (80, 81), but the nature of that importance varies between language groups, nations and clans. There are many aboriginal names for dingoes and other dogs, and some of those names reflect the situation in which they are found, from tamed Type 1 and 2 commensals to completely wild-living animals. For examples, the word “dingo” itself is likely an anglicisation of the Gadigal (a clan of the Eora Nation based near Sydney Cove in Port Jackson) word “tingo” or “tinghu,” which denoted camp dogs (82, 83): the Butchulla/Badjalla people of the Fraser Coats area and the island of K’gari off the Queensland coast refer to island dingoes as “wongari” or “wang’ari,” and to camp dingoes as “wat’dha” (84, 85): “kal” and “wilker/r” are Djadjawurrung words for western Victorian camp dogs and wild-living dingoes, respectively, (86).^2^

The reason or use for the taming of the dingo by Aboriginal communities is difficult to pinpoint. Stories and songlines may have been lost during forceful removal of the people or the recording of differing stories from different groups may have caused confusion as to which is true when they all could be, depending on the location or situation. The evidence available suggests that the tame dingo offered warmth and companionship, particularly for elderly women (87), but very little as a hunting dog (88) except in specific locations (89, 90) where prey is abundant. Some communities used and use them for their ability to recognise supernatural powers and rely on them to know when evil spirits approach (91). Other communities may have collected puppies for the emotive reaction; puppies allow for nurturing in an environment that may not allow for a large human population (92), particularly as these puppies generally returned to the bush once sexually mature (90). Despite the close association with the dingo, they hunted their own food, made their own camp and followed their own lore (80). This changed with the introduction of modern domestic dogs to Australia, which relied on human resources for survival and did not leave the community once mature.

For whatever reason, some dingoes were tamed by Aboriginal people, and they, followed by modern domestic dogs, became critical parts of Aboriginal communities. Although some say dogs are classed as humans (86), most agree that dogs fall somewhere between human and non-human in status (81). Although pups were and are traded, dogs are regarded as family rather than property (93). There are greater consequences for causing harm, or even expressing that you wish to cause harm, to a dog than for any other property (91). Dogs are treated similarly to children; they are taught responsibility and consequence at a young age (94) and are to accompany women not men (95).

In some areas dingoes and other dogs are incorporated into kinship systems (95, 96), whilst elsewhere, in rare circumstances, only certain individuals are (91). Meyers (96) suggests that the inability of dogs to follow kinship lore regarding who one can and cannot marry (mate) is all that separates them from humans. However, unlike humans that suffer severe consequences for breaking such lores, dogs seem to be immune, again concreting their place between humans and nature.

## 4 Public and animal health impacts of Australian free-roaming dogs

### 4.1 Zoonoses

Because dog-related zoonoses is an extensive area of study, we have limited our discussion to a selection pertaining most importantly to Australian free-roaming dogs and One Health approaches to treatment. Free-roaming dogs harbour a range of infectious pathogens (see Supplementary Table S1) that can be variously transmitted to other animals (animal-to-animal zoonoses) and to humans (anthropozoonoses). Many of these pathogens were introduced to Australia through European agricultural pathways and practices after 1788 CE, and continue to cause economic, animal and public health issues (97). Urban sprawl now sees free-roaming dogs not just in rural environments but also peri-urban and urban regions, providing an increased opportunity for the spread of pathogens to not just livestock but to wildlife, pets, and people (98). It is important to understand the public health impacts of these free-roaming dogs and to improve veterinary and public health education to minimise impacts, especially in low socio-economic or remote regions where health services are limited (99). In addition, Australia is continually at threat of the introduction of new, exotic pathogens where free-roaming dogs could be a primary vector for their transmission (97).

#### 4.1.1 Endemic pathogens

Parasitic worms are common in free-roaming dog populations, with tapeworms representing a significant proportion of the overall intestinal pathogens (100). The likely most significant of these is the hydatid tapeworm (*Echinococcus granulosus*). Dogs are the definitive hosts and shed infectious eggs into the environment. These eggs can remain viable for prolonged periods, so long as the environment is not too hot and dry (101). They are consumed by herbivorous intermediate hosts (mostly macropods and domestic livestock) whilst grazing, causing cystic hydatid disease in the liver and lungs of livestock or wildlife. Hydatid infection is invariably fatal for brush-tailed rock-wallabies [*Petrogale penicillata*, (102)], which are vulnerable to extinction. Eggs of *E. granulosus* are sticky and adhere to the fur of infected dogs, passing readily to humans (103). Although hydatid disease is now rare in Australians, it remains a potentially significant pathogen due to the high prevalences in free-roaming dogs in eastern Australia where the majority of the human population resides (98, 104) and has been reported in Aboriginal community free-roaming dogs (105).

Canine hookworms (*Ancylostoma caninum*, *A. ceylanicum*, *A. braziliense* and *Uncinaria stenocephala*) are the most prevalent nematode parasites infecting free-roaming wild dogs (100). Of the four known endemic canine hookworms, *A. caninum* accounts for the majority of infections in Australian dog populations (106) and patent infections in humans are possible (107). Human infection occurs through environmental exposure to hookworm larvae, after they emerge from eggs passed in the faeces of infected dogs. Infective third stage larvae commonly enter humans and other dogs through the skin, via the feet, or by oral exposure to soil contaminated water and foods. Human infection with *A. caninum* can lead to eosinophilic enteritis (108). Human infection in some indigenous communities remains just as prevalent and endemic as it was 40 years ago (109), despite increased awareness, understanding of the lifecycle, and access to health services.

Dog roundworms (*Toxocara canis*) produce thick-walled eggs that are passed in faeces and are highly resistant to environmental conditions (110). These eggs embryonate in the environment, each containing a single second stage larva. The eggs do not hatch until they are ingested by a dog. Once in the dog, the eggs hatch releasing the larvae that develop to an infective third stage and migrate to the lungs, are coughed up and swallowed. Once in the small intestine, the larvae develop through to adults becoming patent in 30–35 days. *Toxocara canis* can also be an important zoonosis. Should eggs of *T. canis* be accidentally ingested by a human [commonly a young child that has a habit of eating soil (pica)] the eggs hatch in the intestine, the released larvae migrate around the body of the child and one may find its way to the back of one of the eyes (111). The larva enters the eye leading to unilateral blindness.

*Strongyloides stercoralis* is a zoonotic intestinal nematode of humans in semi-tropical and tropical areas of Australia (99). Adult parasites infect the intestine, whilst similarly to hookworms, larval stages emerging from eggs passed into the environment with faeces develop into free-living larval stages capable of skin penetration. Free-living larval stages may also develop into adults capable of mating and themselves producing eggs and larvae capable of infecting humans. In addition, *S. stercoralis* can infect dogs. Eggs passed by the worms in infected dogs behave similarly to those passed by humans. They can hatch, release larvae that develop into adult males and females, mate and produce eggs and larvae thus adding to the level of environmental contamination and transmission to humans. Vertical transmission can also occur in pregnant and lactating females infected with hookworms and/or roundworms. This additional pathway can maintain transmission in dog populations not subject to helminth control programs. In the case of hookworms, infective larvae can be transferred to suckling puppies via the mammary glands in the milk and in the case of roundworms, infective larvae migrate from the body tissues of pregnant females to the placenta and infect unborn puppies *in utero,* approximately 2 weeks prior to birth. Some roundworm larvae may also be transferred to suckling puppies via the milk, similarly to hookworms (112).

Heartworm is debilitating and fatal to dogs if untreated in early stages and is globally costly in treatments and preventative medications (113). All Australian free-roaming dogs, including dingoes, are susceptible (114) and prevalence in Type 1 and 2 dogs varies across different climatic regions in association with the distribution of mosquito vectors (115).

Less well understood, is the role of free-roaming dogs in the transmission of bacterial pathogens to other animals and to humans. Despite there being a safe and effective vaccine for Q-fever, many new cases are annually reported across Australia. Historically, human infection was linked to direct contact with infected ruminants, but human infection without direct contact to any animal is increasing (116). Although many species can transmit *Coxiella burnetii*, free-roaming dogs that encroach into urban areas have been detected shedding the bacteria in their faeces which could contribute to cases of human or other species infection (117). Rickettsioses such as *Rickettsia felis*, which is transmitted through fleas, have been detected in Type 1 and 2 community dogs in the Northern Territory (118). Many bacteria exist within the gastrointestinal tract of animals as commensal pathogens. But, exposure to antimicrobial resistant bacteria by wildlife happens through contact with anthropogenic sources (119). This is an extremely understudied field and significantly more information is required to fully understand the role of free-roaming dogs in the transmission of drug-resistant bacteria.

Scabies is a skin irritation associated with poverty that primarily afflicts children in third world countries (120). The pyodermic disease is usually caused by a human-specific mite, *Sarcoptes scabiei* var. *hominis*, but there is evidence that *S. scabiei* var. *canis* that afflicts dogs is linked to scabies in remote Aboriginal communities in the Northern Territory and North Queensland (121, 122). Transmission pathways of scabies infection between Type 1 and 2 commensal dogs, and between dogs and children have only been implied, not measured. In some of those communities, campaigns to treat their dogs, both Type 1 and 2, with oral ivermectin have reduced prevalence of human scabies in the short term (120). A link between interactions of children with Type 1 dogs, childhood scabies, subsequent secondary infections with *Streptococcus pyogenes* and golden staph (*Staphylococcus aureus*) and chronic heart disease and renal infections has long been suspected [e.g., (123)], and treatment of all community dogs through topical or oral acaricide (e.g., ivermectin sandwiches) implemented as a precautionary mitigation (122). The prevalence of rheumatic heart disease, acute rheumatic fever, heart failure and renal failure are disproportionately high among Aboriginal Australians (124, 125), but it will take continued concerted effort to maintain treatment of Type 1 and 2 community dogs and long-term monitoring of disease prevalence to be sure of the relationship. Community differences and sensitivities must be considered because of the high value dogs have for remote communities and a One Health approach could assist.

Not all pathogens harboured by free-roaming dogs have public health significance and there are many with animal health impacts (Supplementary Table S1). One significant, well-studied pathogen is the protozoan parasite *Neospora caninum,* which free-roaming dogs shed (126), and subsequent infection in cattle results in reproduction losses. Transmission in Australia has been reviewed (127) and *N. caninum* infection occurs in Aboriginal community dogs (mostly Types 1, and 2a–2e) and Types 4 and 5 dogs over a wide geographical area (128) and which were likely to have become infected through consuming livestock and/or wildlife. Most recently, viruses of free-roaming dogs were broadly studied by a metatranscriptomic approach, detecting a range of canine important pathogens, including a previously undetected virus (129).

Reverse zoonotic infections (zooanthroponoses), where humans transmit disease to dogs, also occur (130). Humans can transmit pathogens causing bacterial, viral, fungal and parasitic diseases to dogs in domestic situations (i.e., Type 1, 2a, b and c dogs). For example, dogs have been rarely infected with the coronavirus causing COVID-19 (131) and the monkeypox virus (132), and bacterial zooanthroponoses include tuberculosis (133) and golden staph infection (134), and ringworm fungal infections, which particularly affect remote aboriginal community children (135), go both ways. We are not aware of any studies into prevalence or epidemiology of human-to-free-roaming dog populations of such pathogens in Australia.

#### 4.1.2 Exotic pathogens

There are some diseases of canids that are exotic to Australia, but which are concerning because of their potential impacts as all forms of zoonoses if introduced. Canine rabies, which is a fatal, viral zoonosis that remains a significant issue for human health and wildlife management worldwide (136) is the major concern. Terrestrial rabies can be found in most countries and, although it is preventable by vaccination, there is no cure. Annually, 59,000 human deaths from rabies infection are reported, mainly in Asia and Africa where the primary reservoir is the domestic dog (137) and vaccination is not always available. Wildlife and livestock are also affected and provide a reservoir where it is endemic [e.g., raccoons (138, 139)]. Although Australia is currently free of canine rabies, it is spreading through the Indonesian archipelago and increasing the risk to Australian borders. Rabies spread to the eastern Indonesian island of Yamdena (320 km from Australia at their closest points) in 2010, and to Timor Leste (~450 km from the closest part of Australia) in 2023, (140).

If rabies does enter Australia, it has the potential to have a greater social, economic and ecological impact on the continent than any previous incursion of an exotic disease (141). This is because of Australia’s extensive assemblage of susceptible dogs and Australians’ affinity with these animals. Many models have been developed to improve preparedness for a canine rabies outbreak in Australia [e.g., (142–146)]. Model outputs suggest that rabies will progress differently within the functionally different types of dogs. Restrained Type 1 dogs pose limited risk for rabies transmission, because interactions with other dogs are limited and generally supervised by owners. Free-roaming Type 2 (including hunting dogs) will likely play an important role in rabies transmission in some situations, primarily based on their ability to roam, access to other free-roaming dogs and their interactions within and between dog groups (18, 147). For example, northern Australian Indigenous community dogs may play a significant role in the maintenance and spread of rabies in the first instance due to their proximity to a potential incursion, ability to roam freely and their interactions with wild dogs and humans (141, 142, 144). However, Types 2f, 3, 4, 5 and 6 dogs could prove the most critical for rabies spread and maintenance in Australia, because they are widely distributed, often in high abundance, roam over large distances and frequently interact (18). Interactions between Type 2 and Type 3 dogs will also play a pivotal role in rabies transmission, particularly to humans (17, 22). The implementation of effective control strategies for canine rabies in Australia will be reliant on designing programs targeted towards the different types of dogs in Australia, rather than relying on a blanket approach for all dogs and hence a One Health approach will assist.

### 4.2 Predation–death, injury and wellbeing impacts

#### 4.2.1 People

Although dogs are predators, they are not generally considered predators of humans though dogs do kill humans occasionally, and dog attacks on humans are not uncommon (see below). The extreme abundance of dogs globally and their proximity to humans means that injuries, attacks, and some deaths from dogs will inevitably occur. Many urban jurisdictions have dog-keeping laws ultimately intended to reduce these conflicts (see above), and a great amount of management effort is expended on doing so. Dog predation of humans can sometimes occur in relatively rare situations where wild-living dogs become familiar with and habituate to humans and can come to associate them with food. However, attacks on people on K’gari are more associated with familiarisation and habituation than with food availability (148).

There are several potential risks to human health associated with free-roaming dog behaviour, particularly in commensal situations. These dogs can transmit diseases, cause motor vehicle accidents or attack people, including fatally (149). Type 1, 2a and 2b dog attacks, on both humans and other animals, are common and receive media attention across Australia (e.g., see links below). Despite this, no comprehensive nation-wide database records dog attacks (150). Data currently available are dispersed among various government agencies and remains incomplete and inconsistently reported across States and Territories. Information regarding dog attacks on humans is currently collected and recorded by Australia’s health systems (i.e., hospital records), while reports of dogs attacking other animals are generally kept by local government authorities (LGAs). New South Wales is the only state in which councils are legally required to report all dog attacks to the State Government (151). This paucity of information leaves much of the Australian continent vulnerable to contagious disease outbreaks, limiting authorities’ ability to effectively implement management strategies.

During 2024, there were 7,383 dog attacks reported to authorities in NSW. Of those attacks, 44% were on people (*n* = 3,285), with 60% of those attacks resulting in some form of injury, ranging from minor injuries through to those requiring hospitalisation (NSW Office of Local Government, 2025b). The situation of attacks by dogs in Australia-wide data for hospitalisations of people after dog attacks in 2021–2022 was not given in 69% of 9,542 reports, but ~25% were by contained dogs, and ~4.9% were likely free-roaming dogs. However, not all dog attacks are reported, resulting in an underestimation of the true impact dog attacks have on human society (152).

One of the most widely known examples of free-roaming dog/human hazard management in Australia is within the K’gari (Fraser Island) section of the Great Sandy National Park, which is co-managed by the Queensland Parks and Wildlife Service (QPWS) and the Butchulla Aboriginal Corporation (BAC). The 1,640 km^2^ island is a World Heritage Area and a place of ecological and cultural significance supporting a stable isolated population of 100–200 dingoes (mostly Type 2e, 3 and 5 free-roaming dogs) 500 residential dwellings and over 400,000 visitors annually (153, 154).

Free-roaming dogs rarely pose a threat to humans in the wild, but under certain circumstances and with the right provocations, their predatory nature can override wariness of humans, leading to potentially tragic results. Serious cases include the death attributed to wild-living dingoes (Type 5) of Azaria Chamberlain in central Australia (155) and Clinton Gage on K’gari (Type 2e) (156). Between 21st October 2021 and 30th July 2024, 55 high risk interactions with dingoes were reported on K’gari, of which 31 involved people being mouthed or bitten, and 4 people attacked causing multiple bites and punctures, including Clinton Gage and his brother. Concurrently managing risks to human safety and dingo conservation and cultural integrity on K’gari is challenging. The Queensland Government’s Fraser Island Dingo Conservation and Risk Management Strategy [The Strategy, (157)] and associated reviews have built on historic dingo and domestic animal management plans and lessons learned from implementing them to devise strategies to address those competing imperatives (158). The focus of The Strategy, informed by public and stakeholder consultation, has been to break the negative interaction pathway through a range of risk interventions, education, compliance, collaboration and research activities, while acknowledging dingoes are an important component of K’gari’s functioning ecosystems and are protected and conserved as a discrete culturally significant population.

#### 4.2.2 Pets

In rural and peri-urban landscapes it is often the case that wild-living and contained dogs live in close quarters and this presents threats, risks and impacts to both pets and pet owners. In residential areas, wild living dogs will attack and or kill domestic dogs. Although the true cost and consequences of these attacks is difficult to collect, some records are available (151). For example, 4,108 dog attacks on domestic pets were reported to LGAs in NSW during 2024 (Table 1), with 73% of attacks resulting in an injury to the animal, ranging from minor injury (*n* = 775) through to death (*n* = 1,169) and 64% (s.e. = 2.7) of quarterly reports were attacks on other dogs. Although not identified in the data, it is likely that, if attacks on people are representative of attacks on pets, most are caused by Type 1 dogs, but some Type 2 and Type 3 dogs are implicated.

**Table 1:** Quarterly records of dog attacks during 2024 on pets by dogs in NSW (human population size ~8,472,086).

Period	Dog	Cat	Other	Total
Jan–March 2024	634	58	222	914
Apr–Mar 2024	686	69	364	1,119
Jul–Sept 2024	688	64	437	1,189
Oct–Dec 2024	600	68	218	886
Total	2,608	259	1,241	4,108

Such attacks are regularly reported in the media when wildlife are killed and people and pets are attacked.^3^ Often attacks on dogs, especially by wild-living Type 2e and 3 dogs, leave the owners with excessive veterinary bills in treating their pets or are fatal. The psychological effects of dog attacks have not been measured but some very likely have lasting impacts on the mental health of pet owners.

### 4.3 Wildlife–impacts and ecological roles of free–roaming dogs

The ecological roles of free-roaming dogs include predator, competitor, scavenger (159), prey, host and more (160). These roles can be ecologically positive, negative or neutral, but predatory, competitive, and pathogen and ectoparasite host roles have the potential to negatively affect the health and wellbeing of other animals and their populations. Following the extinction of thylacines, recently introduced dingoes became the largest terrestrial non-human predator in Australia (161). But, with flexible habitat requirements, a generalist diet (162, 163) and flexible foraging strategies (13, 164), an average body size of ~ 15.7 kg, dingoes and similarly-sized free-roaming modern dogs are classic mesocarnivores, that elsewhere in other continents would be mesopredators to larger canids, felids and ursids (165, 166). In Australia, they have direct relationships with a wide variety of fauna and flora at different trophic levels: their indirect relationships have been suggested to extend further to soils and soil biota (167).

#### 4.3.1 Threatening process

Although dingoes might have sustainably coexisted with most native Australian fauna for about 5,000 years (168), they did so under pre-British colonisation conditions. Since about 1820, settler exploitation of indigenous country changed the landscapes, including the introduction and exponential multiplication of hard-hoofed ungulate livestock, reduced aboriginal cultural burning, land clearing and habitat fragmentation, artificial water provisioning, altered fire regimes, introduction of invasive herbivores such as rabbits and feral pigs, and increased density of some native fauna like macropods. These conditions predisposed some fauna to increased and unsustainable predation by free-roaming dogs, including dingoes, and European red foxes (*Vulpes vulpes*) and feral cats (*Felis catus*) (169).

The impacts of free-roaming dogs on fauna can be hazardous to the persistence of populations and species (170, 171) and are recognised as a known or potential threat to at least 14 nationally listed species as small as 70 g (172) (Table 2). Some Australian fauna, such as northern hairy-nosed wombats, *Lasiorhinus krefftii* (173), and bridled nail-tail wallabies, *Onychogalea fraenata* (174), are threatened by regional or remote free-roaming dog predation, and greater bilbies are threatened by a combination of remote free-roaming dog and red fox predation (174). In northern Australia, short- and long-term strays, and remote free-roaming dogs are important predators of crocodile and marine turtle eggs, destroying up to 98% of crocodile nests (175) and threatening the survival of endangered turtle rookeries (176). In south-eastern Queensland, the greatest cause of mortality for fragmented koala (*Phascolarctos cinereus*) populations can be peri-urban free-roaming dogs, both dingoes and modern breeds (177, 178), which can threaten local populations with extinction. In semi -arid western New South Wales, regional and remote free-roaming dogs (mostly dingoes) were identified as hazardous to ≤94% of the 80 extant threatened faunal species (179).

**Table 2:** International Union for the Conservation of Nature (IUCN) redlist of threatened species status of populations of some Australian fauna threatened by free-roaming dog predation.

Order	Common name	Scientific name	Adult weight (g)	Redlist status	Likely free-roaming dog type
Aves	Black-breasted button-quail	*Turnix melanogaster*	100	NT	3, 4, 5
Aves	Mallefowl	*Leipoa ocellata*	2,500	V	3, 4, 6
Aves	Southern cassowary	*Casuarius casuarius johnsonii*	60,000	V	2b, 2e, 2g, 3, 5
Mammalia	Southern marsupial mole	*Notorycetes typhlops*	70	E	4, 5, 6
Mammalia	Smoky mouse	*Pseudomys fumeus*	35–65	V	3, 5, 6
Mammalia	Golden bandicoot	*Isoodon auratus*	670	V	2f, 4, 5, 6
Mammalia	Northern quoll	*Dasyurus hallucatus*	1,200	E	2e, 4, 5, 6
Mammalia	Greater bilby	*Macrotis lagotis*	2,500	V	3, 4, 5, 6
Mammalia	Long-footed potoroo	*Potorous longipes*	2,500	E	2c, 2e, 3, 5
Mammalia	Bridled nail-tail wallaby	*Onychogalea fraenata*	8,000	V	3, 4, 5, 6
Mammalia	Proserpine rock-wallaby	*Petrogale persephone*	8,800	E	2c, 2e, 3, 5
Mammalia	Koala	*Phascolarctos cinereus*	12,000	V	2, 3, 5, 6
Mammalia	Northern hairy-nosed wombat	*Lasiorhinus krefftii*	31,000	CE	3, 5, 6
Reptilia	Flatback turtle	*Natator depressus*	90,000	DD (V)	2e, 2c, 2f, 4, 5
Reptilia	Olive ridley turtle	*Lepidochelys olivacea*	46,000	V	2e, 2c, 2f, 4, 5

Refer to text for likely free-roaming dog type. NT = near threatened, V = vulnerable, E = endangered, CE = critically endangered, DD = data deficient.

Being adaptable to prey and scavenge availability, rural and remote free-roaming dogs can switch from preferred species that become less detectable to other, but less-desired species. If those species are threatened by predation and are in low abundance or density, free-roaming dogs can become a threatening process locally. For example, free-roaming dogs in the remote Strzelecki Desert preferentially consume rabbits, *Oryctolagus cuniculus*, but when rabbits become unavailable following drought, disease outbreaks and dust storms, predation rates on dusky hopping-mice (*Notomys fuscus*) increase, threatening their populations (180).

### 4.4 Livestock–negative impacts

Free-roaming dogs can adversely affect livestock in all types of situations where livestock occur, from peri-urban small holdings (by Types 2b, c and e) through to extensive remote cattle stations by Types 4 and 6. The impacts include death and injury (13, 15, 181), disease transmission affecting production and profit [e.g., hydatids (182, 183) and see references above] and stress-related reductions in homeostasis (184). The latter stresses, when occurring during pregnancy, have the potential to affect life-long productivity of offspring (184, 185).

Records of livestock predation losses to all types of free-roaming dogs are inconsistently kept. However, some jurisdictions are making use of the WildDogScan app and website^4^ to geo-spatially record the numbers and types of livestock affected in attacks and monitoring and control efforts. For example, in the Northern Tablelands Local Land Services (NTLLS) district in northeast New South Wales, 684 affected landowners recorded free-roaming dog incidents in WildDogScan from 2020 to 2024 (NTLLS, unpublished data). Reports over the 5 years included killed sheep (*n* = 1,724), lambs (*n* = 484), goats (*n* = 98) and cattle (*n* = 22) and calves (*n* = 76). Injured livestock, requiring veterinary treatment and sometimes euthanasia, totalled 1,383 and annual mean losses were 757.4 livestock (s.e. = 71.3). A few injured horses and alpacas were also recorded. These losses were in the context of concerted annual control efforts to reduce Type 4 free-roaming dog populations living adjacent to livestock holdings and did not include peri-urban losses to Type 2 free-roaming dogs.

There are also contrary contributions about neutral and positive effects of Type 4 free-roaming dogs. These include; the failure to detect benefit of controlling Type 4 dingoes on calving percentages in South Australia (186), potential benefits to grazing pressure by large macropods in semi-arid environments (187), and claims of increased cattle production when Type 4 dingoes are present (188).

The psycho-social impacts of predation by free-roaming dogs on livestock can be extreme and cannot be underestimated (189). Predation intruded on owners’ lives, as indicated by persistent thoughts about dog attack, anger and frustration particularly with government agencies, sleep deprivation, and negative impacts on personal relationships (189). The level of distress and psychological impact on individuals was equivalent to those experienced by survivors of major vehicle accidents and partners of breast cancer victims, and second only to Vietnam/USA War veterans with PTSD (189).

## 5 Strategic adaptive management cycle

**Figure 4:**
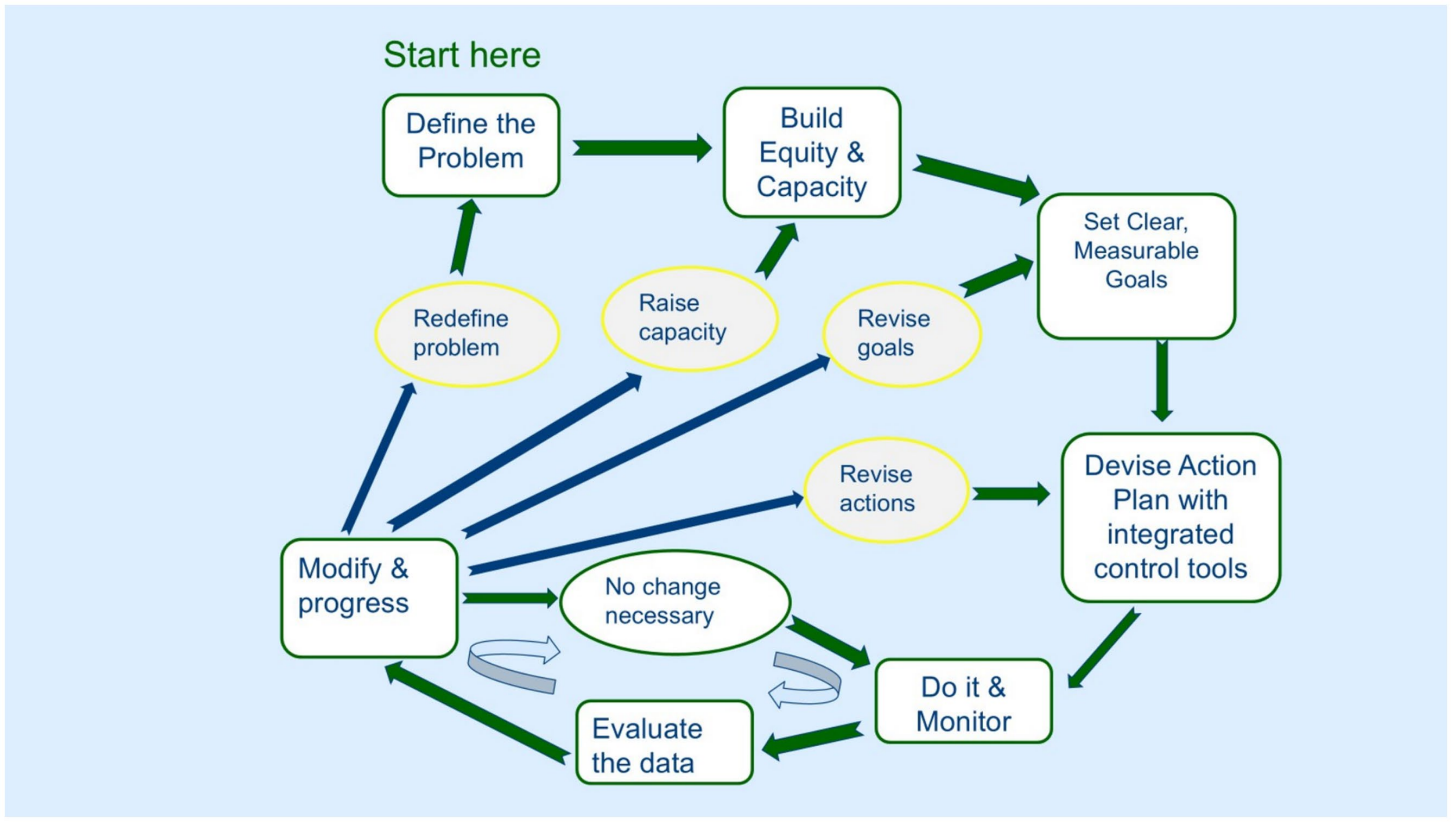
A strategic management cycle showing the seven required sequential steps (green boxes linked by green arrows), the sub-cycle where no changes are necessary (pale blue arrows) and where changes can be made after data evaluation (blue arrows) [adapted from (41)].

The strategic approach to managing invasive animal impacts (13, 40, 190) very much applies to the complex and wicked problems caused by free-roaming dogs in Australia. It is a version of passive adaptive management, or “learning by doing” (191), and entails systematic acquisition & application of reliable information to management of any issue, but particularly to issues with landscape scale and multiple stakeholders. It assists with issues where there is insufficient issue definition; accidental or deliberate exclusion of key stakeholder/s; incapacity of stakeholders through lack of knowledge, time or funding; inability to work together because stakeholders are unused to working in groups, have traditional adversarial behaviour between stakeholders; or stakeholders have conflicting objectives and obligations. There are seven steps in the strategic management cycle (Figure 4):

1. Define the issue quantitatively and qualitatively, with baseline measurements of impacts, and identification of the types of dogs involved; where the problem occurs and at what scale; when, how and how often it occurs; who the stakeholders are including those directly and indirectly involved; and what the drivers and barriers to adoption are;
2. Build equity and capacity among stakeholders, which involves a threshold level of knowledge, time and funding;
3. From the data provided, set clear measurable goals;
4. Devise a plan of actions that identifies who will be doing what, when and by when;
5. Implement the plan and monitor everything that is relevant to assessing the relative success of the plan;
6. Evaluate the resultant monitoring data, and;
7. Revise the plan based on the evaluation, and then move to the next iteration as results indicate.

An advantage of this approach is that it allows for co-development of plans and evaluations, and for changes to be made where they are needed or where actions can be improved given new knowledge and data. The cycle shows where an operational plan can fail and where to fix it. Plan iteration begins with no change being necessary, increases in effort in the action revision section, and then occurs with increasing difficulty back to redefining the problem. It is an active adaptive approach (192) that has been used successfully for implementing free-roaming dog management [e.g., (193)].

Environmental psychology methods can be used to define the human dimensions prior to implementation of the One Health and One Welfare approaches. Those measurements can be used to segment the audience and devise interventions to change behaviours where necessary and to set goals for changes in behaviour. Adoption rates can be measured and revised interventions can be iteratively implemented to increase adoption.

### 5.1 Framework for a One Health approach

A One Health framework fits well within a strategic adaptive management approach, by first defining the issue from many perspectives, identifying awareness and knowledge gaps, developing strategies to raise community awareness of those issues as they pertain to their health and wellbeing and those of their animals and communities, collecting the specialists and community stakeholders required to implement the strategies, putting monitoring procedures in place, evaluating outcomes of any interventions and suggesting solutions to shortcomings, and iteratively revising and reimplementing plans. Problem definition for free-roaming dog management in a One Health framework seeks to provide a cohesive, evidence-based pathway that balances ecological integrity, human wellbeing, cultural values and social license. Successful implementation will require sustained commitment, coordinated interdisciplinary collaboration, and culturally respectful communications and engagement across all sectors of society to build the collective capacity needed to manage this ‘wicked problem’.

The One health High Level Expert Panel (OHHLEP) outlines pathways for change under the One Health Joint Plan of Action model, including: equitable and inclusive governance and sustainable financing, integrated data and surveillance, and transdisciplinary capacity building (194) Informed by this model, effective dog management must:

- Include both Indigenous and non-indigenous peri-urban, regional and remote stakeholder voices;
- Link wildlife (environmental), domestic animal, livestock and human health surveillance systems;
- Be appropriately resourced with stable funding and technical capacity, and;
- Promote ongoing cross-sectoral coordination, collaboration and capacity building.

Current *governance* mechanisms are fragmented across jurisdictions and sectors. The establishment of a national, multi-agency taskforce, that increases the scope of the National Wild Dog Action Plan to coordinate representatives from human health, animal health, wildlife ecology, Indigenous communities, and local governments to unify policy goals, harmonise data collection and surveillance systems, will coordinate funding and facilitate rapid response to emerging risks.

Integrating companion animal, livestock, and wildlife health *data and surveillance* capabilities is essential for early detection of zoonotic threats. Data on movement ecology and contact rates and environmental impacts need to be measured and shared across all relevant agencies.

#### 5.1.1 Socio-cultural engagement

Public attitudes to dogs vary markedly across Australia. Audience segmentation and behavioural insights can help tailor communication strategies to peri-urban, regional, remote, and Indigenous audiences (195). Understanding stakeholder and public sentiment is fundamental to shaping effective behaviour change strategies. Respectful inclusion and utilising co-design processes to develop culturally appropriate interventions with all impacted stakeholders, including Indigenous and remote communities including will improve the effectiveness and legitimacy of place-based decision-making and management actions (196–198).

#### 5.1.2 Ecological management

Ecologically-informed management must distinguish between commensal and rural wild-living, big mover dog populations and remote area wild-living and flexible populations of dogs. Where the negative impacts of all types of dog on livestock production through zoonoses and predation, behavioural ecology can be used to target lethal or exclusion management. Where culturally appropriate and generally desired or legislated, the dingo component of Types 4, 5 and 6 free-roaming dogs can be managed for conservation. Neutering programs (199) can be successful in reducing commensal free-roaming dog population size and impacts if sensitively implemented with adequate community engagement and buy-in. Examples include those implemented in responsible pet ownership programs and those undertaken by Animal Management in Rural and Remote Indigenous Communities (AMRRIC) in remote and regional communities with large commensal dog populations. However, trap-neuter-release programs are unlikely to work long-term in the open populations that predominate in Australia because of the wide distribution of Type 4, 5 and 6 dogs (Figure 1). Further integration of research on free-roaming dog genetics, ecological roles, and biodiversity impacts, and benefits and costs of free-roaming dog population control and conservation strategies will assist in increasing the knowledge base and potentially reduce conflict (200).

#### 5.1.3 Adaptive policy and funding

Interpreting differing cultural attitudes, beliefs, world views and human–animal relationships to shape socially informed policy is essential and environmental psychology approaches are valuable in ascertaining what those are. Policies must remain flexible to accommodate adaptive management principles and refinement based on scientific and field evidence and stakeholder feedback (34). Stable funding and institutional commitment are needed to support long-term monitoring and intervention (201).

To implement a One Health framework regional priorities, existing programs, and gaps will need to be scoped and mapped as part of stakeholder network mapping activity (see Problem Definition in the Strategic Approach above). Integrated dog management programs need to continue to be trialled in high-priority areas such as remote Indigenous communities or biodiversity hotspots, with adaptive management cycles informed by robust monitoring and evaluation using both quantitative outcomes (e.g., disease prevalence, dog density) and qualitative metrics (e.g., community satisfaction) robust monitoring and evaluation. Successful models and outcomes will require ongoing funding, training, and legislative support for long-term sustainable outcomes.

#### 5.1.4 Stakeholders and disciplines involved in a One Health approach

Operationalising a One Health framework for managing the impacts of free-roaming dogs in Australia will require investment in cross-sector partnerships, culturally inclusive governance, and robust evidence-based integration. There are diverse stakeholders who can be united under shared goals for health, sustainability, and social harmony. One Welfare embraces the same objectives but specifically adds a focus on wellbeing and welfare of animals and people.

**Figure 5:**
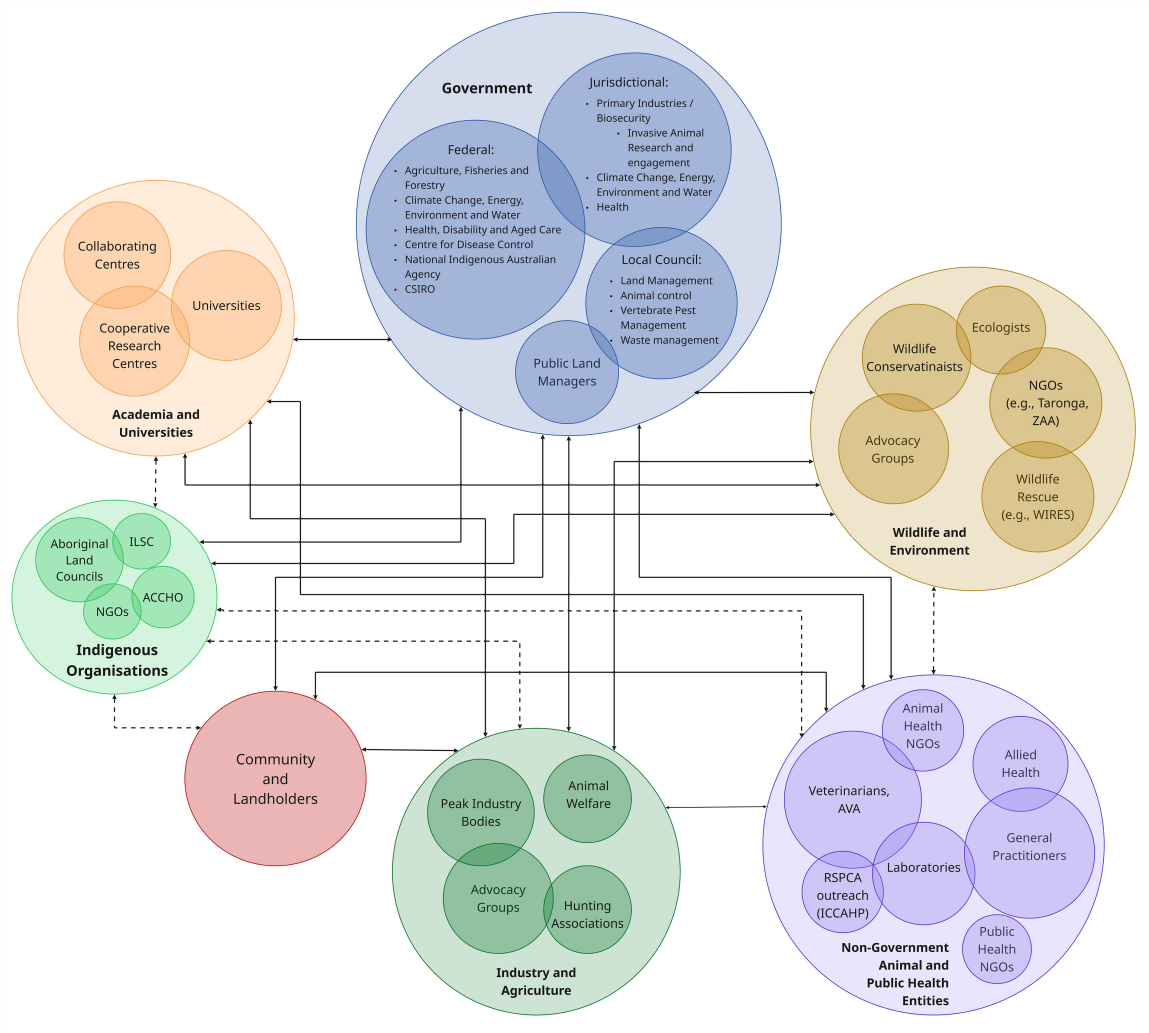
Stakeholders who are either impacted or have a role to play in the management of free-roaming dogs, and their interactions. The thickness of the connectors denotes the strength of the relationship.

Effective implementation of a One Health framework necessitates collaboration across multiple sectors and disciplines. The complexity of managing free-roaming dogs, which entwines public health and welfare, animal health and welfare, environmental conservation, different cultural norms and cultural heritage, requires coordinated action among six stakeholder groups (Figure 5), at least three of which are key:

Government, including:

- Australian (Commonwealth/federal) and State and Territory (jurisdictional) agencies: including the Department of Agriculture, Fisheries and Forestry (DAFF); Department of Health, Disability and Aged Care (DoHDAC), Department of Climate Change, Energy, Environment and Water (DCCEEW), National Indigenous Australians Association (NIAA) and their State and Territory counterparts, and;
- Local Government Authorities and Natural Resource Management agencies and bodies that are responsible for managing national park estate, invasive animal biosecurity matters across-tenures, domestic animal control, community safety, and waste management.

Industry, including:

- Animal and Public Health Practitioners: such as veterinarians and para-veterinarians, general practitioners, allied health professionals and laboratory staff who overseeing zoonotic disease surveillance, vaccination programs, and human and companion animal health;
- Industry and Agricultural Sectors: Particularly livestock producers and primary industry peak bodies and associations impacted by dog predation and biosecurity risks;
- Wildlife Conservationists and Ecologists: Managing biodiversity impacts and assessing the ecological roles of all free-roaming dog types;
- Animal Welfare and Rescue Organisations: Involved in sterilisation, sheltering, rehoming, and promoting responsible pet ownership and rehabilitating impacted wildlife species, and;
- Academic and Research Institutions: Including government invasive animal research institutions, universities, collaborating centres and research cooperatives who conduct interdisciplinary research in ecology, epidemiology, behaviour, social license and policy.

Community, including:

- Indigenous Organisations and Traditional Owners: Custodians of land and cultural knowledge with active involvement in flexible dog population research and community-based remote area and wild living dog management, and;
- Community Members and Landholders: Both peri-urban, regional and remote residents and communities affected by and influential in local free-roaming dog management strategies.

The proposed transdisciplinary and multi-stakeholder model for free-roaming dog management aligns with the WHO One Health Theory of Change, which emphasises cross-sectoral inclusivity, equity, and systemic integration (202).

## 6 Barriers and drivers to adoption

To measure the adoption and outcomes of a One Health approach to managing free-roaming dog impacts requires that a qualitative and quantitative assessment of community attitudes, beliefs, world view and knowledge is undertaken [e.g., (38, 39)]. The environmental psychology approach we outlined enables the determination of the characteristics of adoption by key stakeholders and provides direction to overcoming barriers and for enhancing drivers. In addition to psycho-social and other human dimensions data, a One Health/One Welfare strategic approach to management requires essential indigenous and other cultural information, human and veterinary health data (particularly spatial data on zoonoses and dog attacks), livestock production and processing loss data (e.g., predation, hydatids and dog bites) and ecological data.

## 7 Conclusions and recommendations

The strategic adaptive management approach provides a framework in which to apply environmental psychology and achieve the behavioural changes required for the application of One Health to free-roaming dogs in Australia. Assuming there is a willingness to improve the management of free-roaming dogs in a One Health context, capacity to change or implement management strategies requires relevant knowledge, sufficient time and investment.

The first step is to define the issue from the perspectives we have outlined, particularly from the human behaviour viewpoint. Investment in measuring the current knowledge, demographics, beliefs, values and behaviours of stakeholders is essential. To facilitate the necessary collaboration between human and animal health professionals, livestock husbandry personnel, ecologists and ethologists, and veterinary pathologists, animal carers and veterinarians requires that each discipline appreciates the interconnectedness among affected disciplines. Once a benchmark is established, we need to raise awareness about current and potential health and welfare issues incurred from and by free-roaming dogs in Australia and the associated complex interactions of culture, epidemiology and ecology.

Adoption requires that One Health stakeholders are aware that there is an issue that affects or potentially affects them, are educated in the details that are relevant to them, and are incentivised to adopt change through behaviour change marketing techniques such as community-based social marketing (203, 204). This will require political will and investment to acquire required knowledge, facilitate necessary collaborations, undertake qualitative and quantitative social research, determine and apply the social marketing interventions to address the research results. It is crucial that the impacts of behaviour change on the public and animal health, welfare and wellbeing, and the economics of free-roaming dog impacts are monitored over time and evaluated to assess whether the required behaviour changes have been achieved.

## Footnotes

^1^We use the term “feral” as defined by Charles Darwin (i.e., an animal derived by a process of domestication that has gone wild, and its descendants) in the glossary of his seminal work 13 (205).

^2^Out of respect to indigenous Australians’ usage conventions and protocols, we have only used indigenous names where they are in the public domain.

^3^E.g., https://www.newsofthearea.com.au/irresponsible-pet-ownership-in-the-spotlight-after-vicious-attack-on-nambucca-senior, https://www.abc.net.au/news/2025-03-27/nsw-dog-ownership-review-after-fatal-attacks/105021692.

^4^ https://feralscan.org.au/wilddogscan
