# Supplementary material for: Diverse human dimensions affect the management of public and animal health impacts of free-roaming dogs in Australia: a One Health solution
Source: Front Vet Sci. 2025 Oct 23;12:1666111. doi: 10.3389/fvets.2025.1666111 (PMC12590507; doi:10.3389/fvets.2025.1666111)
Supplement: Supplementary file 1 [file Table_1.DOCX]

| **Pathogen** | **Prevalence** | **Impact** | **Reference** |
| --- | --- | --- | --- |
| **Cestodes:** | | | |
| *Dipylidium caninum* | medium | low | Brown and Copeman 2003) |
| *Echinococcus granulosus* | High | High | Jenkins et al 2008  (Jenkins, Lievaart et al. 2014), Harriott, Gentle et al. (2019) |
| **Nematodes:** | | | |
| *Ancylostoma braziliense* | low |  | (Smout, Thompson et al. 2013) |
| *Ancylostoma caninum* | medium - high | low | (Smout, Thompson et al. 2013) |
| *Ancylostoma ceylanicum* | high* |  | (Smout, Thompson et al. 2013) |
| *Dirofilara immitis* | low |  | Brown and Copeman (2003) |
| *Strongyloides stercoralis* | low | low | Beknazarova, Whiley et al (2020 |
| *Toxocara canis* | low |  | Jenkins (2020) |
| *Trichuris vulpis* | low | low | Stevenson and Hughes (1988) |
| *Uncinaria stenocephala* | low |  | (Harriott, Gentle et al. 2019) |
| **Pentastomes:** | | | |
| *Linguatula serrata* | low |  | (Shamsi, McSpadden et al. 2017, Birckhead, Jenkins et al. 2024) |
| **Bacteria:** | | | |
| *Salmonella spp.* | Low |  | (Harriott, Wood et al. 2019) |
| *Brucella suis* | Nil^ - low ^#^ |  | (Harriott, Wood et al. 2019, Kneipp, Sawford et al. 2021) |
| *Campylobacter spp.* | Nil^ |  | (Harriott, Wood et al. 2019) |
| *Coxiella burnetii* | medium |  | (Shapiro, Brown et al. 2017, Harriott, Wood et al. 2019) |
| MSSA | Low |  | (Harriott, Wood et al. 2019) |
| MRSA | Low |  | (Rusdi, Laird et al. 2018) |
| *drug resistant E. coli* | medium |  | (Rusdi, Laird et al. 2018, Harriott, Wood et al. 2019) |
| Rickettsia spp. |  |  | (Hii, Kopp et al. 2011, Bennett, Abdad et al. 2017, Harriott, Wood et al. 2019) |
| Anaplasma spp |  |  | (Constable, Brown et al. 2008, Shapiro, Brown et al. 2017) |
| **Protozoa:** | | | |
| Giardia | low |  | (Rusdi, Laird et al. 2018) |
| Cryptosporidia spp | low |  | Ref? May be a problem in immune compromised people |
| *Neospora caninum* | low |  | Davidson, Huaman et al (2022) |
| *Isospora spp*. | low |  | (Rusdi, Laird et al. 2018) |
| *Sarcocystis spp.* | low |  | (Rusdi, Laird et al. 2018) |
| **Viruses:** | | | |
| Various | low | low | (Mifsud, Harvey et al. 2025) |

*localised – we recognise that some pathogens have varying prevalence specific to localised regions and are not widespread across the country. This table represents the reported values in publications even if their prevalence is location specific and prevalence may be impacted by specific environmental factors.

^Nil recordings do not mean that the pathogen is absent form free-roaming dog populations, just that they were tested for, but not detected in sample.

^#^ Data from domestic population (pig dogs) that have had exposure to wild pigs

**Prevalence scale – average prevalence across studies**

Low – 0-20%

Medium – 20.1 – 50%

High – 50.1 – 100%

**Impact based on? – prevalence, known spread, presence in other species, potential for human disease**

Unknown (not enough information)

Low

Medium

High
